# Therapeutic Potentials of Microalgae in the Treatment of Alzheimer’s Disease

**DOI:** 10.3390/molecules22030480

**Published:** 2017-03-18

**Authors:** Tosin A. Olasehinde, Ademola O. Olaniran, Anthony I. Okoh

**Affiliations:** 1 Applied Environmental Microbiology Research Group, Department of Biochemistry and Microbiology, University of Fort Hare, Alice 5700, Eastern Cape, South Africa; aokoh@ufh.ac.za; 2 SAMRC, Microbial Water Quality Monitoring Centre, University of Fort Hare, Alice 5700, Eastern Cape, South Africa; 3 Nutrition and Toxicology Division, Food Technology Department, Federal Institute of Industrial Research Oshodi, PMB 21023, Lagos Nigeria; 4 Department of Microbiology, School of Life Sciences, College of Agriculture, Engineering and Science, University of KwaZulu-Natal, Private Bag X54001, Durban 4000, South Africa; olanirana@ukzn.ac.za

**Keywords:** Alzheimer’s disease, microalgae, bioactive compounds, neuroprotection, oxidative stress, cholinesterases, β-secretase

## Abstract

Current research is geared towards the discovery of new compounds with strong neuroprotective potential and few or no side effects compared to synthetic drugs. This review focuses on the potentials of extracts and biologically active compounds derived from microalgal biomass for the treatment and management of Alzheimer’s disease (AD). Microalgal research has gained much attention recently due to its contribution to the production of renewable fuels and the ability of alga cells to produce several secondary metabolites such as carotenoids, polyphenols, sterols, polyunsaturated fatty acids and polysaccharides. These compounds exhibit several pharmacological activities and possess neuroprotective potential. The pathogenesis of Alzheimer’s disease (AD) involves complex mechanisms that are associated with oxidative stress, cholinergic dysfunction, neuronal damage, protein misfolding and aggregation. The antioxidant, anticholinesterase activities as well as the inhibitory effects of some bioactive compounds from microalgae extracts on β-amyloid aggregation and neuronal death are discussed extensively. Phytochemical compounds from microalgae are used as pharmaceuticals, nutraceuticals and food supplements, and may possess neuroprotective potentials that are relevant to the management and/or treatment of AD.

## 1. Introduction

Alzheimer’s disease (AD) is a neurological disorder which occurs via complex pathophysiological mechanisms, resulting in memory loss, cognitive impairment, neuronal death, loss of synapses and cholinergic neurons, brain damage and ultimately, cell death [1,2]. It is the most common form of dementia. The morbidity and mortality rate of patients suffering from AD is increasing in both developed and developing countries of the world. A recent report revealed that more than 46 million people are living with dementia, and this figure is expected to rise to above 130 million by 2050 [3]. Although the etiology of AD is not fully understood, previous reports have shown that multiple factors such as oxidative stress, low levels of acetylcholine, accumulation of metals, β-amyloid aggregation and loss of synaptic neurons contribute to its development [4,5,6]. Current pharmacological strategies towards the treatment and management of AD involve the use of cholinesterase and β-secretase (BACE-1) inhibitors [5,7]. Rasagline, rivastigmine and donepezil are common cholinesterase inhibitors which increase acetylcholine levels in the AD brain. However, these drugs exhibit side effects, such as nausea, vomiting, dizziness, hepatoxicity and gastrointestinal disorders [8]. Moreover, synthetic BACE-1 inhibitors are still under clinical trials [7,9]. Although BACE-1 inhibitors prevent β-amyloid aggregation in AD patients, some of these drugs which are currently being developed have been dropped due to their toxic nature, and it is rather too early to discover whether any of these agents could be effective in the management of AD [7]. Furthermore, most of the drugs used in the management of AD are single targets. Some multifunctional synthetic agents have shown less pharmacological activity in vivo due to poor absorption, toxicity and inability to pass through the brain barrier. Since the onset of AD involves different pathological mechanisms, biologically active compounds with multiple targets could be more effective in the management of AD [10]. Polidori [11] reported that there is a strong association between poor nutrition and Alzheimer’s disease. Hence the use of functional foods, dietary supplements and nutraceuticals could be an effective therapeutic intervention in the management of AD. Recent experimental investigations have focused on the use of dietary supplements and/or natural products with multi-neuroprotective activities which pose little or no side effects compared to synthetic drugs.

Studies involving the use of microalgae have gained much interest due to their potential health benefits. The great diversity with respect to several species and strains of microalgae combined with their phytochemical constituents could give a clue to the huge interest these organisms have generated in the search for various compounds useful for the treatment of chronic diseases. Nutritional studies have also revealed that the incorporation of a microalgal biomass and/or its bioactive compounds in foods and beverages may reduce the risk of dementia and prevent memory loss in AD patients. Microalgae are excellent sources of various biologically active compounds such as sterols, fatty acids, phenolic compounds, carotenoids and polysaccharides [12]. Previous reports have revealed the anti-inflammatory [13], anticancer [14], hypocholesterolemic [15], antioxidant [16] and antiviral [17] activities of microalgal-derived extracts and compounds.

This review focuses on the neuroprotective activities of extracts and biologically active compounds derived from microalgae which are associated with radical scavenging activity, inhibition of metal-induced lipid peroxidation, anti-cholinesterase activity, anti-amyloid aggregation and β-secretase activity.

## 2. Microalgae as Potential Sources of Biologically Active Compounds

Microalgae are unicellular, microscopic and photosynthetic microorganisms that grow in fresh or salt water. Although most microalgae are eukaryotes, some still exist as prokaryotes. Cyanobacteria are prokaryotes and are mostly classified as microalgae [18,19]. There are about 800,000 species of microalgae that exist in different forms. However, about 35, 000 species have been reported in the literature [20,21]. Most microalgae live in wild and unpleasant environmental conditions and can divide rapidly due to their unicellular and simple multicellular structure. As a result of their unique metabolism, microalgae react to changes in their external environment with changes in their intracellular environment. During the growth stage of most microalgae, different metabolic processes in the cells lead to the biosynthesis of various compounds and molecules. Microalgae are a reservoir of primary and secondary metabolites which are released during different growth stages in algal cells.

Because of the presence of several primary and secondary metabolites in algal cells, microalgal biotechnology has received much interest, with its application in the energy, food, pharmaceutical and cosmetic industries. Microalgal biomass are used as energy source to produce biodiesel, bioethanol and biohydrogen due to their high growth rate and their lipid and carbohydrate contents [22,23]. Algal biomass from *Dunaliella* sp., *Nannochloropsis* sp. and *Scenedesmus* sp. have been used to produce biofuel and aquafeeds [24]. Apart from the application of *Scenedesmus* sp. and *Nannochloropsis* sp. in biofuel production, these two microalgae are also utilized to produce nutraceuticals due to their fatty acid, vitamin and mineral compositions. *Dunaliella* sp. are rich in carotenoids and xanthophyls and are used as food and feed additives. A previous report has revealed the application of some microalgal biomass in the development of nutritional supplements due to their health benefits [24]. *Spirulina* sp. and *Chlorella* sp. are also sources of nutraceuticals such as proteins, vitamins, minerals, polysaccharides and polyunsaturated fatty acids (PUFAs) [24].

*Chlorella* sp. is significantly known for its phycocyanin content and *Chlorella* growth factor (CGF) which is a water-soluble extract, contains a mixture of proteins, vitamins, nucleic acids, sugars, essential amino acids and peptides [25]. *Chlorella* sp., *Arthrospira* sp. and *Aphanizomenon flos-aquae* have been produced commercially as nutraceuticals and food supplements in form of tablets, capsules and liquids [26]. Some edible strains have been incorporated into beverages, pasta and snack foods either as food additives or nutritional supplements [26]. *Chlorella* and *Spirulina* are utilized and processed into microalgal foods such as microalgal noodles, bread, drinks, biscuits, green tea, candy and beer [27]. Raposo et al. [28] reported that algal biomass from some microalgae prevents cardiovascular diseases and strokes and these could be linked to their sterol and PUFA contents. A study carried out by El-Baky et al. [29] revealed that *Dunaliella* supplements rich in carotenoids exhibited antihyperlipidemic activities. *Arthrospira* has good nutritive value and is commercially packaged as tablets and powder. Experimental investigations on *Arthrospira* sp. revealed that it can attenuate hyperlipidemia, hypertension and reduce serum blood glucose [26]. *Spirulina maxima* is now referred to as *Arthrospira maxima* because of its current taxonomical classification. It is used as a food supplement and has been shown to exhibit hypolipidemic effects and reduce blood pressure in human subjects [30]. Furthermore, dietary supplementation with *S. maxima* also reduced ischemic brain damage and prevented lead-induced kidney and liver damage [31,32].

The use of dried algal biomass and algal-derived biologically active compounds as pharmaceuticals has received much attention recently. [33,34,35]. Polysaccharides from microalgae are potent immunomodulatory, anti-inflammatory, hypocholesterolemic, hypolipidemic and hypoglycemic agents [28,36]. Sulfolipids have been identified and isolated from some microalgae: *Scenedesmus rubescens* [37], *Scenedesmus acuminatus* [37], *Phaeodactylum tricornutum* [38] and *Pavlova lutheri* [39]. These compounds have been discovered to be potent inhibitors of glutaminyl cyclase, α-glucosidase and telomerase activities [37]. Sulfolipids also exhibit several biological activities, such as anti-inflammatory, anti-neoplastic, antiviral, immunosuppressive and anti-proliferative activities [37]. Biologically active compounds derived from different species of microalgae have been shown to possess several pharmacological properties such as anticoagulant, anti-inflammatory, anticancer, antimicrobial, antioxidant and antiviral activities [33,34,35]. Different classes of compounds such as carotenoids, phenolic compounds, polyunsaturated fatty acids, polysaccharides, sterols, phycobiliproteins, vitamins and alkaloids have been identified in various microalgal strains.

### 2.1. Carotenoids

Carotenoids are natural pigments with high relevance in food and pharmaceutical industries. Carotenoids are synthesized in microalgal cells and they contribute to their photosynthetic organelles, thereby protecting these cells against photo damage. More than 600 carotenoids have been reported, however, the common types that are present in human diets include β-carotene, lycopene, lutein and cryptoxanthin [40]. Studies have reported the carotenoid contents of different strains of microalgae [41,42]. Previous reports have also shown that carotenoids present in fruits and vegetables can prevent coronary heart diseases, cancer and other degenerative diseases [43,44]. Some microalgal strains are capable of synthesizing natural isomers of β-carotene. For example, *Dunaliella salina* has been reported to release up to 10–13% dry weight of β-carotene into its biomass [45]. Other carotenoids such as astaxanthin, canthaxanthin, and lutein have been identified in *Haematococcus pluvialis* [46,47], *Chlorella zofingiensis* [48] and *Arthrospira* sp. [46]. Carotenoids contribute to the antioxidant capacity of some microalgae [41,49]. They also play a major role in the prevention of chronic diseases [40].

### 2.2. Phenolic Compounds

Polyphenols are the most commonly occurring natural compounds and are present in diverse plants [50]. Previous reports have revealed the pharmacological activities of different classes of phenolic compounds from different plants [51,52,53]. Phenolic acids such as gallic, chlorogenic and ferulic acids are known for their high antioxidant activities [54,55,56]. Oboh et al. [53] reported that gallic and chlorogenic acids exhibit cholinesterase inhibitory activities and protect the brain against metal-induced lipid peroxidation. Phenolic compounds are synthesized during secondary metabolism in some algal cells [57]. However, not all microalgal strains can synthesize phenolic compounds. Flavonoids were identified in methanol, ethanol and chloroform extract of *Desmococcus olivaceus* and *Chlorella vulgaris* [58]. Machu et al. [59] reported the phenolic content of the aqueous and methanol extract of *Chlorella pyrenoidosa* (18.0 and 25.8 mg/g gallic acid equivalent respectively) and *Spirulina platensis* (43.2 and 24.8 mg/g GAE, respectively). In the same study, high-performance liquid chromatography (HPLC) analysis revealed the presence of gallic acid, 4-hydroxybenzoic acid and epigallocatechin in *C. pyrenoidosa* while catechin, epicatechin and pyrocatechol were found in *S. platensis* [59]. Safafar et al. [60] identified gallic acid, ferulic acid and cinnamic acid in algal biomass derived from *Nannochloropsis salina* and *Nannochloropsis limnetica*. Furthermore, the HPLC profile of phenolic extract from *Spirulina maxima* revealed the presence of phenolic acids (chlorogenic, gallic, ferulic, coumaric, vanillin and cinnamic acids) and flavonoids (quercetin, genistein, kaempferol, pinostrobin and galangin) [61].

### 2.3. Polyunsaturated Fatty Acids

Polyunsaturated fatty acids (PUFAs) especially omega-3 and omega-6 PUFAs, are essential fatty acids which cannot be synthesized in human cells but are produced by microalgae cells during their growth stages. Polyunsaturated fatty acids are predominant in the human brain and are important in neurological processes involving synaptic transmission, cognitive development, memory function and neuronal plasticity [62,63]. Poor intake and deficiency of omega-3 fatty acids in the brain may lead to cognitive impairment, memory dysfunction and neuronal damage [64]. Imbalance of omega-3 and omega-6 fatty acids may lead to symptoms associated with AD [62]. There are indications that omega-3 PUFAs modulate learning and memory function in the brain [63]. Docosahexanoic acid (DHA) is the most important PUFA in the brain [65]. The brain contains elevated levels of omega-6 PUFA such as arachidonic acid (AA) and DHA but low levels of eicosapentanoic acid (EPA) [66]. Endogenous synthesis of these fatty acids is limited in the human brain; however, the brain regulates levels of PUFAs via dietary intake since they can easily cross the blood–brain barrier via simple diffusion [67]. Microalgae are an excellent reservoir of omega-3 PUFA [68]. Data from previous reports revealed that some microalgae (*Arthrospira* sp., *Isochrysis* sp., *Odontella* sp., *Pavlova* sp., and *Porphyridium* sp.) produced high amounts of DHA and EPA [47,69]. *Crypthecodinium cohnii* is predominantly rich in DHA producing up to 40–50% with the absence of EPA [70]. *Nannochloropsis oculata*, *Phaeodactylum tricornutum* and *Porphyridium cruentum* are also rich in EPA [71,72].

### 2.4. Polysaccharides

Research into the biological activities of polysaccharides and their applications in biomedical and pharmaceutical industries have attracted much interest recently. Polysaccharides are the most predominant form of carbohydrates in nature and they occur mostly in plants. Polysaccharides such as fucoidan, laminaran, carrageenan, agar, alginate and proteoglycans have been identified in some algae [73]. There is increasing attention on algal-derived polysaccharides because of their marked biological activities. Sulfated polysaccharide has been identified in brown algae and has been reported to prevent neuronal damage and cell death in PC12 neuronal cells [74]. Glucans (α and β) have the capacity to improve hippocampal synaptic plasticity [75]. Previous reports indicate that polysaccharides contribute to the antioxidant activities of some microalgae. Some glucanic polysaccharides possess strong antioxidant activities [76]. Polysaccharides derived from marine *Isochrysis galbana* are potent scavengers of hydroxyl and super oxide radicals [77]. Polysaccharides isolated from *Pavlova viridis* and *Sarcinochrysis marina* are also potent scavengers of hydroxyl radicals and inhibitors of lipid peroxidation [78]. Polysaccharides derived from *Spirulina platensis* showed protective effects against neuronal damage [79]. The immunomodulatory, anticancer, anti-inflammatory and anticoagulant activities of some alga-derived polysaccharides have been reported [80,81,82,83]. However, to the best of our knowledge the neuroprotective activities of alga-derived polysaccharides have not been fully explored.

### 2.5. Sterols

Sterols are naturally occurring cholesterol-like triterpenes which are dominant in plants. Sterols are similar to cholesterol with respect to their structure and function. However, cholesterols are present in animals and rarely occur in plants. Phytosterols contribute to the regulation of membrane fluidity and permeability and form part of the structural components of cell membrane in different organisms [84]. Apart from plants and macroalgae, microalgae are excellent sources of novel phytosterols with several biological activities. Recently, Ahmed et al. [85] reported that *Nannochloropsis* sp. BR2, *Pavlova lutheri*, and *Tetraselmis* sp. M8 contain high levels of phytosterols, generating up to 0.4–2.6% dry weight from the algal biomass. Some phytosterols precisely ergosterol and 7-dehydroporiferasterol were extracted from *Dunaliella tertiolecta* and identified via silver ion liquid chromatography [86]. Similarly, ergosterol, 7-dehydroporiferasterol, ergosterol peroxide and 7-oxocholesterol have been identified in *Chlorella vulgaris* [87]. Stigmasterol, sitosterol and fucosterol have also been confirmed in *Chrysoderma* sp., *Chrysomeris* sp. and *Chrysowaernella* sp. [88]. Among several microalgae, isofucosterol has been identified in *Chattonella antiqua*, *Chattonella marina*, *Chattonella subsalsa* [89], 24-ethylcholesta-5,22-dien-3β-ol, campesterol and sitosterol in *Cyanophora paradoxa* [90], campesterol, sitosterol, and clionasterol in *Nostoc commune var. sphaeroides Kützing* [91].

The use of phytosterols in food and pharmaceutical industries has gained huge interest recently due to their biological activities and their functional properties. Phytosterols exhibit anti-inflammatory [92], anticancer [93], antidiabetic [94], antihypercholesterolemic [95] and antioxidant [96] activities. Recent data have indicated that sterols may play an important role in improving neurological functions and prevent neuroinflammation in the central nervous system [97,98]. The ability of phytosterols to easily cross the blood brain barrier could facilitate their role in improving cognition in AD patients [86]. Despite the diverse biological activities of phytosterols, there are limited reports on the neuroprotective activities of microalgal-derived phytosterols.

## 3. Neuroprotective Potentials of Microalgae against Alzheimer’s Disease

### 3.1. Targeting Oxidative Stress

Oxidative stress has been implicated in the development and pathogenesis of AD. Brain cells are predisposed to free radical attack due to their high lipid content, low levels of antioxidant enzymes and the inability of their neurons to synthesize glutathione [4,99]. Overproduction of reactive oxygen species (ROS) in brain cells can lead to free radical attack against PUFAs at the cell membrane which can in turn induce lipid peroxidation [100]. Lipid peroxidation products (4-hydroxynonenal and malondialdehyde) are very toxic to neurons and can induce neuronal death [101]. Furthermore, some redox metals can also contribute to the pathogenesis of AD. Aluminium and iron act as catalysts for ROS production due to their loosely bound outer electron and capacity to exist in more than one valence. Iron can induce the breakdown of hydrogen peroxide to form hydroxyl radicals [102]. Hydroxyl radicals are very reactive in nature and can abstract electron from PUFAs to form a lipid radical which can induce lipid peroxidation [102].

Several reports have established that free radical scavenging mechanisms, metal chelation and inhibition of metal-induced lipid peroxidation in the brain are therapeutic targets for the management of AD [100,103]. Antioxidants are known to be radical scavengers, metal chelators and inhibitors of lipid peroxidation. Ataie et al. [104] reported that antioxidants derived from natural products can prevent neuronal damage and progression of AD. Some microalgae have been reported to possess antioxidant compounds which could play a neuroprotective role via the prevention of nerve cell injury and inhibition of radical-induced neuronal damage [72]. *Chlorella vulgaris* extract attenuated oxidative stress induced cell death [105]. *Spirulina* and *Chlorella* water extracts also scavenged 2,2'-azino-bis-3-ethylbenzothiazoline-6-sulphonic acid (ABTS) and 2,2-diphenyl-1-picrylhydrazyl (DPPH) radicals [106]. *Chlorella Vulgaris* attenuated lead-induced oxidative damage in rats’ brains via increase in superoxide dismutase (SOD), catalase (CAT), glutathione peroxidase (GPX) and glutathione (GSH) levels as well as reduction of malondialdehyde (MDA) levels [107]. Li et al. [49] reported that microalgae *Synechococcus* sp. FACHB283, *Chlamydomonas nivalis* and *Nostoc ellipsosporum* CCAP 1453/17 possess strong antioxidant capacities and thus could be potential rich sources of natural antioxidants. *C. vulgaris*, *H. pluvialis*, *N. oculata*, *Neochloris oleoabundans*, and *Chaetoceros calcitrans* extracts scavenged ABTS radicals, exhibited ferric reducing antioxidant activity and prevented lipid oxidation in vitro [41]. The antioxidant activities of each microalgal strain were linked to their carotenoid and phenolic content.

In another study, the antioxidant activity of *H. pluvialis* extract was attributed to its carotenoids (astaxanthin) and fatty acid (oleic acid, myristic and palmitic acids) contents [108]. Furthermore, methanol and hexane extracts of four different microalgae (*Tetraselmis chuii*, *Nannochloropsis oculata*, *Chlorella minutissima* and *Rhodomonas salina*) scavenged DPPH radicals and were able to chelate metal ions (iron and copper) [72]. The hexane extract displayed stronger scavenging and chelating activity than the methanol extract. The higher scavenging and chelating ability of the hexane extract was attributed to PUFAs, which were abundant in the extract. The study also revealed that sterols and phenolic compounds such as tocopherol and butyl hydroxyl toluene also contributed to the scavenging and chelating abilities of the extract. Hydroxyl radical scavenging activities of aqueous and methanol extract of *Chlorella stigmatophora* and *Phaeodactylum tricornutum* indicate their protective effects against lipid peroxidation and neuronal damage [13].

Carotenoids display strong antioxidant activities and this can be linked to their chemical structures. β-carotene, lutein and lycopene exert their antioxidant activity via the quenching of radicals and singlet oxygen [109]. The antioxidant activity of most carotenoids has been linked to the multiple conjugated doubled bonds and energy transfer between electrophilic singlet oxygen and their polyene backbone [110]. The radical scavenging activity of lutein is linked to its polarity, hydroxyl groups and multiple conjugated double bonds. Culture cell studies have shown that lutein exhibits stronger antioxidant activity via inhibition of lipid peroxidation and attenuation of oxidative damage compared to β-carotene [111]. The antioxidant activity of lutein could be linked to the additional hydroxyl groups present in the structure. Astaxanthin showed neuroprotective activity via inhibition of hydrogen peroxide-induced neuronal damage [112]. Apart from the radical scavenging activity of astaxanthin, cellular antioxidant assays have shown that this carotenoid increases cellular glutathione levels, restores endogenous SOD and inhibits malondialdehyde (MDA) production [113]. Astaxanthin prevents neuronal injury and cell death via activation of protein kinase B (Akt) and extracellular signal-regulated protein kinase-1/2 (ERK1/2) pathways [114]. Wu et al. [115] demonstrated that astaxanthin triggered the expression of glutathione-S-transferase-α1 (GST-α1), quinone oxidoreductase (NQO-1) and heme oxygenase (HO-1) via the nuclear factor erythroid-related factor 2 and the antioxidant responsive element (Nrf2-ARE) pathway (Nrf2-ARE) signaling pathway and inhibited oxidative stress. Furthermore, astaxanthin attenuated oxidative damage and alleviated brain aging by restoring GSH, SOD and GPX activities and decreasing MDA, protein carbonylation and DNA adducts in rats’ brains [116]. Xu et al. [117] also reported that astaxanthin suppressed oxidative stress and improved cognitive function via the down regulation of caspase 3/9 expression and the upregulation of phosphoinositol-3 kinase and protein kinase B (PI3KT/AKT) expression in diabetic rats.

The antioxidant activity of microalgae could also be linked to some phenolic compounds. Safafar et al. [60] reported that coumaric, gallic and caffeic acids contributed to the antioxidant activities of *Chlorella* sp., *Nannochloropsis* sp., *Dunaliella* sp. and *Phaeodactylum* sp. *Spirulina maxima* extract contains phenolic acids and exhibits antioxidant protection in rats’ brain homogenates [118]. Phenolic extracts derived from *S. maxima* also scavenged hydroxyl radicals and inhibited lipid peroxidation [61]. The antioxidant activities of the extract could be linked to the presence of phenolic compounds such as kaempferol, quercetin coumaric, ferulic, gallic acid and chlorogenic acid. Phenolic acids and flavonoids are potent scavengers of hydroxyl radicals, metal chelators and inhibitors of Fe^2+^- induced lipid peroxidation in rats’ brains [103].

### 3.2. Targeting Cholinesterase Activity

Clinical evidence suggests that the inhibition of acetylcholinesterase (AChE) and butyrylcholinesterase (BChE) activities are effective therapeutic targets for the management of AD [119,120]. Elevated cholinesterase (ChE) activities degrade acetylcholine (ACh) rapidly thereby causing cholinergic deficit and memory impairment in AD patients [121]. Synthetic drugs inhibit AChE and BChE; thereby increasing ACh levels in the synaptic nerves. ACh is a neurotransmitter which helps to transmit nerve impulses across the neurons. Elevated levels of acetylcholine attenuate cholinergic deficits and improve cognition and memory function [122]. Apart from increasing ACh levels, cholinesterase inhibitors (ChEi) also prevent β-amyloid induced neuronal death by modulating α-secretase activity which acts on amyloid precursor protein, thereby inhibiting β-amyloid aggregation [123]. Rivastigmine, galathanmine and donepezil are synthetic drugs used to improve cholinergic deficits in AD patients. However, these drugs confer several side effects [124]. Furthermore, apart from rivastigmine, other synthetic drugs can only inhibit AChE [121,125]. Selective inhibition of BChE is an effective strategy in the management of AD. Histochemical studies have revealed that BChE is present in some cholinergic neurons in which AChE is absent [126]. In addition, BChE can induce neurotoxicity of some plaques which may contribute to the progression of AD [127].

Extracts from some green algae have potential inhibitory effects on ChE activity and could possibly improve cholinergic transmission in AD patients [128]. Hexane and methanol extracts from *N. oculata*, *C. minutissima*, *Tetraselmis chuii* and *R. salina* extracts inhibit AChE activity in vitro [72]. Similarly, methanol extracts from some microalgal species (*Nannochloropsis* sp., *Picochlorum* sp. and *Desmochloris* sp.) exhibit inhibitory effects on BChE activity but are not potent on AChE activity [129]. Custodio et al. [72] found that *N. oculata* and *Botryococcus braunii* extracts exhibited potent AChE and moderate BChE inhibitory activities.

Furthermore, some bioactive compounds which have been identified in algae extracts exhibit dual inhibitory effects on AChE and BChE. Recent reports revealed that chlorogenic acids, gallic acids, quercetin, rutin and quercitrin, which were identified in *Heinsia crinita* and *Clerodendum volubile* contribute to their anticholinesterase inhibitory activities in vitro [103,130]. Similarly, caffeic and chlorogenic acids also displayed potent inhibitory effects on AChE and BChE activities [53]. There is a dearth of information on the cholinesterase inhibitory effects of phenolic compounds derived from microalgal extracts. Moreover, phenolic compounds with AChE and BChE inhibitory potentials have been identified in *Picochlorum* sp. and *Nannochloropsis* sp. Anticholinesterase activities of some microalgal extracts could also be linked to their carotenoid contents. Although there are few or no reports on cholinesterase inhibitory potentials of carotenoids, isomers of lutein and zeaxanthin have been reported to inhibit AChE activity [131].

Studies have shown that PUFAs exhibit neuroprotective activity and can improve neurotransmission in cholinergic neurons [132,133]. However, there is no evidence of their inhibitory effects on cholinesterases. Poor intake and deficiency of omega-3 PUFAs have been associated with the early onset of AD [134]. Diets supplemented with omega-3 PUFAs can replace the membrane fluidity and prevent oxidative damage to neurons [135]. Microalgae are very rich in PUFAs such as DHA and EHA. Although, there are few reports on the inhibitory effects of PUFAs on AChE and BChE activity, a previous report has shown that they exhibit neuroprotective potential by modulating cholinergic activity and enhancing transmission of nerve impulse in cholinergic neurons [133]. Lesa et al. [136] reported that long chain fatty acids improved neurotransmission in *Caenorhabditis elegans*. Furthermore, a DHA-supplemented diet restored membrane content and enhanced the release of acetylcholine in rats’ hippocampus [137,138]. Lauritzen et al. [132] demonstrated that PUFAs exhibited neuroprotective activity via inhibition of abnormal synaptic transmission and neuronal death in neuronal cultures.

### 3.3. Targeting β-amyloid Aggregation

AD is characterized by loss of synapsis, neuronal death and cognitive dysfunction due to the deposition of senile plaques and neurofibrillary tangles which are induced by β-amyloid (Aβ) peptide [139]. β-amyloid is produced from the proteolytic cleavage of amyloid precursor protein (APP) by β-secretase and γ-secretase [140]. γ-secretase is also referred to as preselinin proteins while β-secretase is a transmembrane aspartate protease which is commonly referred to as β-site APP cleaving enzyme (BACE-1) [141]. BACE-1 has been identified as an alternative drug target for the treatment of AD. This enzyme cleaves APP into soluble APPb (sAPPb) and C-99 peptide. γ-secretase subsequently cleaves the C-99 peptide to form Aβ-40 and Aβ-42 [142]. Overproduction of β-amyloid triggers the misfolding of proteins that is observed in the AD brain which include β-amyloid aggregation, formation of senile plaques and neurofibrillary tangles around the neurons [139]. β-amyloid aggregation mediates the early events in the pathogenesis of AD, and this includes the loss of neurons, neuronal death, neuroinflammation and oxidative stress [143,144]. Therefore, decreasing β-amyloid production and inhibition of β-amyloid-aggregation are important therapeutic strategies involved in the treatment and management of AD.

Furthermore, the inhibition of BACE-1 activity is critical in preventing the overproduction of Aβ and Aβ-induced neurotoxicity [142]. BACE-1 is very important in the formation of Aβ, therefore it is considered to contribute significantly to cognitive dysfunction and memory loss which are associated with AD. BACE-1 inhibitors possess potential application in the treatment of AD. Currently, some synthetic BACE-1 inhibitors are under clinical trials [7]. However, plant extracts and their bioactive constituents have been reported to reduce Aβ-production, inhibit Aβ- aggregation and Aβ-mediated neuronal death in experimental rats’ brains and neuronal cells [145,146,147]. Several reports have revealed the role of macroalgae extracts and their compounds against β -amyloid aggregation and the decrease in β-secretase activity.

There are limited reports on the protective effects of microalgal extracts against β-secretase inhibition and Aβ-aggregation. *Nannochloropsis ocaenia* extract protected neuronal cells against Aβ-induced toxicity [148]. *N. ocaenia* extract also inhibited Aβ-induced oxidative stress and elevated SOD, GSH and CAT levels in neuro-2A neuroblastoma cells. The anti-amyloid and antioxidant activities of the algal extract was attributed to DHA and EPA. Several studies with epidemiological evidence have shown that omega-3 PUFA reduces the risk of AD [149,150]. Chronic administration of DHA improved cognition and learning ability in β-amyloid infused rats [151]. The ameliorative effect of DHA is linked to synaptosomal membrane fluidity [152]. A study performed by Lim et al. [150] suggests that DHA prevents β-amyloid production and aggregation via the modulation of APP processing and reduction of both alpha- and β-APP C-terminal fragment products and full-length APP. In addition, DHA supplementation inhibited β-amyloid aggregation via modulation of fibrillar oligomer in APP/PS1 transgenic rats’ brains [153]. Minogue et al. [154] also reported that EPA inhibited β-amyloid production in the hippocampus of rats.

Apart from omega-3 PUFAs, other bioactive compounds which have been identified in microalgae extract have also shown protective effects against Aβ-induced neurotoxicity. Biochanin A is an isoflavone with estrogenic activity in humans and has been identified in *C. vulgaris* [155]. Biochanin A exhibits neuroprotective activity against Aβ-induced neurotoxicity in PC-12 cells [156]. Furthermore, some carotenoids also protect the neurons against β-amyloid-induced neurotoxicity. Astaxanthin, canthaxanthin and β-carotene showed neuroprotective effects against Aβ25–35-induced cell death in undifferentiated PC12 cells [157]. In a study conducted by Chang et al. [158], astanxanthin was found to protect neuronal cells against apoptotic cell death induced by Aβ25–35 via inhibition of radical induced oxidative damage, inhibition of Bax expression and nuclear transcription factor kB (NFkB) as well as the suppression of phosphorylation of P-38 mitogen activated protein kinase (MAPK). This report also correlates with the investigation of Wang et al. [159] which revealed that astaxanthin protected SH-SY5Y cells against β-amyloid-induced cell death via induction of heme oxygenase-1 expression.

## 4. Future Perspectives

The precise mechanisms by which microalgal-derived bioactive compounds and extracts exert their neuroprotective activities are not fully understood, however, recent findings show that the neuroprotective effects of microalgal-derived compounds and extracts are the result of their antioxidant activities, inhibition of cholinesterases, protection against β-amyloid aggregation and neuronal damage. The neuroprotective activities exhibited by some microalgal strains are mediated by carotenoids, PUFAs, phenolic compounds and sterols.

Most findings reported so far are based on the in vitro antioxidant and anticholinesterase activities of microalga-derived extracts and products; although there are few reports on the inhibition of β-amyloid aggregation via elevation of heme-1 oxygenase expression, modulation of APP processing and Aβ-fibrillar oligomer. However, there are no available data on the inhibitory effects of microalgal extracts on β-secretase and γ-secretase activities. These enzymes are the major targets of synthetic drugs which are under clinical trials for the treatment of AD. There is insufficient evidence regarding the in vivo inhibitory effects of microalgae extracts on cholinesterases and their ability to improve cholinergic transmission as well as memory function in animal models. No detailed reports have been published that determine the effect of microalgal administration on cognitive impairment and emotional disturbances using the maze water and open field test in animal models. To the best of our knowledge, there are limited or no reports on the use of microalgae extracts as BACE inhibitors in animal models and evidence from clinical studies are not available. Research investigation into the bioactive compounds derived from microalgae is a promising research area for the treatment and management of AD.

Several studies have established the promising roles of bioactive molecules from some microalgae in the treatment of cardiovascular diseases, diabetes and inflammation. The neuroprotective activities of most microalgal extracts have been attributed to bioactive compounds such as carotenoids, PUFAs, sterols and phenolic compounds. However, there are few or no reports on the neuroprotective effects of alkaloids and phenolic compounds derived from microalgae. There are insufficient reports from animal models and clinical studies to validate and confirm the established in vitro reports. It is therefore important that future experimental investigations focus on in vitro, in vivo, and clinical studies. Furthermore, there should be emphasis on the effects of some specific bioactive compounds (alkaloids, phenolic compounds, sterols and carotenoids) isolated from microalgae on different pathological targets involved in the development and progression of AD.

## 5. Conclusions

There is growing evidence that microalgal-derived bioactive compounds and extracts may improve cholinergic function, antioxidant status and prevent memory impairment as well as neuronal injury in the AD brain. Some microalgal extracts and their chemical constituents such as carotenoids, phenolic acids, flavonoids, sterols and polyunsaturated fatty acids exhibit promising neuroprotective activities that are relevant to the treatment and management of AD. These compounds and extracts exhibit strong antioxidant, anticholinesterase and anti-β-amyloid aggregation activities. Microalgae species extracts such as those of *C. vulgaris*, *H. pluvialis*, *N. oculata*, *N. oleoabundans*, and *C. calcitrans* are rich in carotenoids and protect the brain against neuronal damage and/or cell death associated with oxidative stress. *N. oculata*, *C. minutissima*, *T. chuii* and *R. salina* extracts possess strong inhibitory effects on AChE activity and could be explored as potent AChE inhibitors. DHA and EPA isolated from *Nannochloropsis ocaenia* appear to be good bioactive molecules with neuroprotective activities via inhibition of Aβ-induced oxidative stress in the AD brain. Astanxathin and Biochanin A are promising therapeutic agents and protect the brain against β-amyloid-induced neurotoxicity and neuronal damage. This carotenoid and isoflavone could be explored for nutraceuticals and pharmaceuticals for the treatment of AD. The additive and synergistic effects of the chemical constituents of most algal extracts may contribute immensely to their neuroprotective activities rather than their singular action. However, the neuroprotective effects of microalgal-derived compounds and extracts in animal and clinical studies involving AD are under-researched. Future studies should focus on investigating the neuroprotective potentials of specific compounds in some microalgal biomasses and determine their effects on the pathological mechanisms linked with AD in animal and clinical models.
